# Cross-Sectional Associations between Playing Sports or Electronic Games in Leisure Time and Life Satisfaction in 12-Year-Old Children from the European Union

**DOI:** 10.3390/ejihpe12080075

**Published:** 2022-08-13

**Authors:** Diego Gomez-Baya, Hugo Sarmento, Javier Augusto Nicoletti, Francisco Jose Garcia-Moro

**Affiliations:** 1 Department of Social, Developmental and Educational Psychology, Universidad de Huelva, 21007 Huelva, Spain; 2 University of Coimbra, Research Unit for Sport and Physical Activity (CIDAF), Faculty of Sport Sciences and Physical Education, 3040-248 Coimbra, Portugal; 3 Department of Humanities and Social Sciences, Universidad Nacional de La Matanza, San Justo B1754, Argentina

**Keywords:** sport practice, electronic games, leisure time, life satisfaction, children, Europe, cross-sectional

## Abstract

Leisure time activities in childhood may have a crucial role in the development of subjective well-being. Nevertheless, more research is needed with cross-national samples concerning the differential effects of lifestyles on life satisfaction. The aim of this cross-sectional study was to analyze the associations between the frequency of playing sports/exercise or playing electronic games on life satisfaction in a sample of 12-year-old children from nine countries from the European Union. The data used in this publication come from the third wave of the Children’s Worlds project, an international survey of children’s lives and well-being whose administration started in 2017. The sample was composed of 10,626 children (50.9% boys) from Estonia, Belgium, Croatia, Hungary, Italy, Malta, Poland, Romania, and Spain. They completed the Student Life Satisfaction Scale and answered two questions to assess the frequency of playing sport or exercise, and the frequency of playing electronic games. The results indicated notable scores in life satisfaction in all participating countries. The results showed some differences between boys and girls, and among the countries, in the frequency of sport practice and electronic games in the leisure time, as well as in the overall level of life satisfaction. The results also underlined that sport practice had a greater positive effect on life satisfaction than the use of electronic games. Thus, this study highlights the need to design programs to promote sport practice in leisure time for 12-year-old children in Europe, in order to protect their subjective well-being.

## 1. Introduction

The importance of health is beyond doubt in today’s society, and health and wellness models are considered an important goal to be achieved and maintained throughout life [1]. The different actions in this sense are implemented transversally throughout the entire life cycle of the person because their beneficial effects are indisputable. The available scientific evidence shows the importance of health, well-being, leisure, and free time for the positive development of the person throughout his or her development [2,3,4,5,6]. However, it is necessary to continue the research on the impact of different lifestyles and their repercussion on happiness and the use of free and leisure time of the child population. In doing so, the ultimate aim is to prevent situations that negatively affect the normal development of the child and promote those leisure time activities that lead to a solid consolidation of attitudes and habits that materialize in a state of personal satisfaction.

Regarding this and central to our research is the concept of subjective well-being, which refers to a person’s subjective evaluation of the quality of his or her life [7]. Its study comprises the scientific analysis of how people evaluate their life in a specific or generic way [8], and it is especially interesting from the point of view of a positive vision towards human development. We can define leisure as the set of free and gratifying experiences that take place in free time, where emotions, motivations, values, benefits, or perceptions acquire great protagonism [9,10]. In this sense, leisure is fundamental for the development of the person. Lifestyle in leisure time refers to the way of life of an individual or population group, which is influenced by personal characteristics and individual behavior patterns, as well as by general living conditions and sociocultural aspects [11], and does not refer exclusively to physical health, but also considers mental health, giving a relevant role to social and emotional intelligence [12].

What actions performed in children’s free time and leisure time lead them to perceive greater personal satisfaction? It seems that interpersonal situations that lead to sharing or spending time with others favor the feeling of happiness. Time spent with others, the acts of generosity and the dynamics of helping and sharing during physical activity with others contribute to greater happiness than those behaviors aimed at obtaining material goals [13,14]. Many hours spent in front of a screen have a negative influence on sports activities and physical exercise [15]. Other contributions showed that the simple fact of having free time is a source of personal satisfaction [16]. More specifically, it seems that activities related to physical activity and sport are considered pleasurable for children [1].

Concerning the most cited areas for subjective well-being, reported by 11 and 12-year-old children, results have underlined such aspects as family, leisure, friendship, school, and receiving gifts [16]. Regarding leisure time, it has been shown that physical activity is an element that enhances psychological well-being, producing an increase in self-esteem, a more positive self-concept, and improving cognitive functioning, as well as some important benefits in social relationships [17]. According to the WHO definition, physical activity can be defined as “any bodily movement produced by skeletal muscles that requires energy expenditure and can be performed at a variety of intensities, as part of work, domestic chores, transportation or during leisure time, or when participating in exercise or sports activities” [18] (p. 15). Strong evidence indicates that physical activity in children and adolescents confers the following health related issues: improved cardiorespiratory and muscular fitness, reduced adiposity, better cardiometabolic and bone health, better cognitive functioning (i.e., better academic performance and improved executive function), and reduced depressive symptoms [18]. The WHO recommends at least 60 min a day of physical activity to develop these positive outcomes, as well as advising a limit to the amount of sedentary time, especially recreational screen time [18], taking into account that sedentary lifestyles gradually increase with age while physical activity decreases, which is detrimental to people’s health [19]. The international Health Behavior in School-aged Children (HBSC) study conducted in 42 countries in Europe and North America showed that approximately 63% of adolescents exceeded screen time recommendations [20], meaning that daily tasks involving the company of others are being reduced [21,22]. In this sense, adolescents recognize that they spend less time doing leisure activities such as playing sports [23].

Further research is needed to examine the differential effects of active vs. sedentary leisure time activities on adolescent subjective well-being in a cross-national manner. The analysis of these effects in different countries in the European Union could provide some ideas to motivate policy design for strengthening adolescent well-being.

The aim of the present study is to analyze the life satisfaction of 12-year-old adolescents in relation to their leisure time; specifically, whether they give more priority to sport and physical activity or to being in front of a computer/screen. The data used in this publication come from the third wave of the Children’s Worlds project, an international survey of children’s lives and well-being. We expected that the practice of sport or exercise in leisure time would be more strongly associated with higher life satisfaction than the frequency of playing electronic games.

This research aims to provide cross-national evidence in some countries from the European Union with representative samples of girls and boys just before the transition to Secondary education. This period is especially important in the development of healthy lifestyles in subsequent years. As well, this research is important because it aims to contribute to the development of policies that promote active leisure activities that favor subjective well-being and quality of life in school-age children based on evidence [24].

## 2. Materials and Methods

### 2.1. Participants and Data Collection Procedure

The data used in this publication come from the third wave of the Children’s Worlds project, an international survey of children’s lives and well-being (www.isciweb.org, accessed on 1 August 2022). The views expressed here are those of the author(s), not necessarily those of ISCWeB. Children’s Worlds is world-wide research into subjective wellbeing in childhood and its determinants. This project’s purpose is increasing awareness about the need to improve psychological wellbeing in children, in order to influence government policies and services around the world. This survey collected information regarding subjective well-being, daily activities, and developmental contexts in children aged 8, 10 or 12 years old. Specifically, the present work analyzed data from 12-year-old children from the nine participating countries from the European Union, with our variables of interest. We chose the European Union sample in order to examine children whose developmental contexts and culture are more similar, as well as aiming to provide some insights from policy design in these regions. This age is included in early adolescence, which is a crucial stage in the development of lifestyles and psychological well-being, while facing puberal changes and encountering changes in social relationships, and just before the transition to Secondary education. A cross-sectional and descriptive study was conducted in these nine European countries. Data collection was carried out throughout 2017 by administering an online self-report measure to a representative sample of children in the selected schools. All the enrolled students were invited to participate in the study. Ethical approval was obtained in each country, as well as informed consent from parents and adolescents. Participants’ anonymity and confidentiality were respected.

The sample was composed of 10,626 children (50.9% boys) from nine European Union countries: Belgium (Flemish) (10.1%); Croatia (10.9%); Estonia (10.2%); Hungary (9.4%); Italy (Liguria) (11.1%); Malta (7.1%); Poland (10.9%); Romania (10.8%); and Spain (Catalonia) (19.6%). Concerning cohabitation, 98.3% lived with their families, and 92.2% of the sample reported that they always slept in the same house.

### 2.2. Measures

Life satisfaction. A questionnaire based on the Student Life Satisfaction Scale, developed by Huebner [25] was used. The scale is composed of six items (“I enjoy my life”; “My life is going well”; “I have a good life”; “The things that happen in my life are excellent”; “I like my life”; and “I am happy with my life”), using an 11-point Likert scale from 0 “Not at all agree” to 10 “Totally agree”. This scale presents excellent internal consistency reliability [21] with α = 0.955. A confirmatory factor analysis revealed a good factorial validity, χ^2^(9) = 1612.73, *p* < 0.001, CFI = 0.976, SRMS = 0.016, with all the indicators showing factor loading over 0.65. A mean score from the initial four indicators was calculated to perform the analyses in this study, with scores ranging 0 and 10 and a theoretical average of 5, with 10 indicating high life satisfaction.

Frequency of playing sport or doing exercise. The participants were asked “How often do you spend time playing sports or doing exercise?”, and six response options were presented: 0, “Never”; 1, “Less than once a week”; 2, “Once or twice a week”; 3, “Three or four days a week”; 4, “Five or six days a week”; and 5, “Every day”.

Frequency of playing online games. The participants were asked “How often do you spend time playing electronic games (on a computer or other device)?”, and six response options were presented: 0, “Never”; 1, “Less than once a week”; 2, “Once or twice a week”; 3, “Three or four days a week”; 4, “Five or six days a week”; and 5, “Every day”.

### 2.3. Statistical Analyses

First, a percentage distribution of the frequency of leisure sport and exercise, and the frequency of playing electronic games were examined, in the total sample and by country and gender. The Kolmogorov–Smirnov test showed that our variables did not follow a normal distribution (life satisfaction: KS = 0.24, *p* < 0.001; leisure sport and exercise: KS = 0.16, *p* < 0.001; computer use: KS = 0.23, *p* < 0.001). Differences by country and gender in these leisure time activities were examined with Χ^2^ tests. Moreover, descriptive statistics of life satisfaction (mean and standard deviation) were calculated, also in the total sample and by country and gender. Non-parametric tests (i.e., Mann–Whitney and Kruskal–Wallis tests) were performed to examine differences by gender and country in life satisfaction.

Second, Kruskal–Wallis (KW) tests were performed to examine differences in life satisfaction based on the frequency of playing sports or electronic games. Then, linear regression analyses were conducted to examine the joint effects on life satisfaction by the frequency of playing sport and the frequency of playing electronic games, by gender and country. Standardized coefficients and explained variance were described.

Third, a new variable was calculated based on the comparative frequency of playing sports and electronic games. Both variables were transformed in dichotomic variables (high level: five or more times a week; low level: less than five times a week), and then a variable was created based on the levels in both habits (low level in playing sports and electronic games; low level in playing sports and high level in electronic games; high level in sports and low level in electronic games; high levels in both sports and electronic games). Kruskal–Wallis tests were conducted to analyze the effect of this variable on life satisfaction. The significance level was set at *p* < 0.05. Data analyses were performed with the software JASP 0.16.1.0 (University of Amsterdan, Amsterdam, The Netherlands).

## 3. Results

### 3.1. Descriptive Statistics

**Figure 1:**
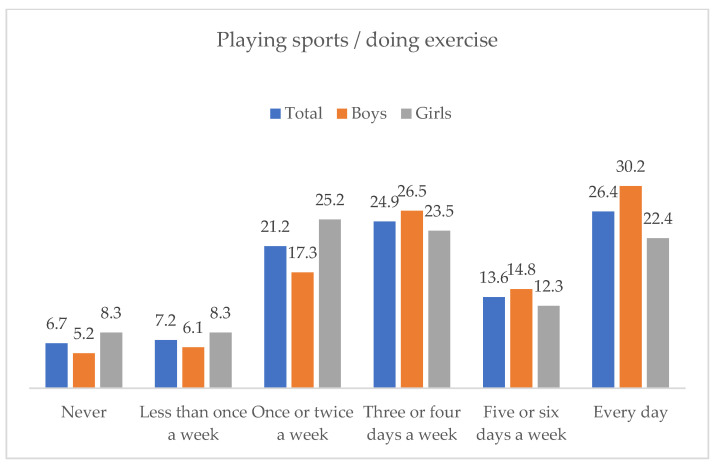
Frequency of playing sports or doing exercise, in the total sample and by gender.

Figure 1 describes the frequency distribution of playing sports or doing exercise. The results indicated that 24.9% of the sample practiced sports three or four days a week and 26.4% practiced it every day. Some gender differences were detected, so that boys reported more frequent sport practice than girls, Χ^2^(5, *N* = 10,184) = 208.12, *p* < 0.001, φ = 0.143. Thus, 30.2% of the boys played sports every day, compared to 22.4% of girls.

**Figure 2:**
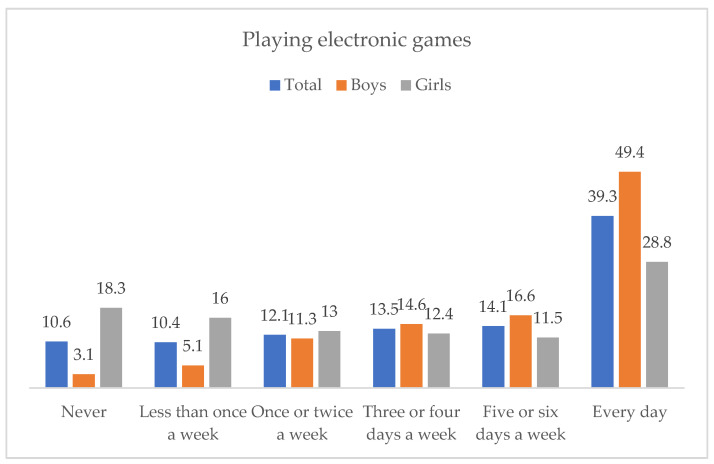
Frequency of playing electronic games, in the total sample and by gender.

Figure 2 presents the frequency distribution of playing electronic games. Around 40% of the sample reported that they played electronic games every day. Some gender differences were detected, Χ^2^(5, *N* = 10,261) = 1195.32, *p* < 0.001, φ = 0.341, so that 49.4% of the boys played electronic games every day, while this percentage was 28.8% among girls.

Furthermore, Table 1 presents the means and standard deviations in life satisfaction, by gender and country. Notable mean scores were found in life satisfaction, with an overall mean of 8.70 (SD = 1.80), in a range from 0 to 10. Some gender differences were detected in life satisfaction, Mann–Whitney U = 8.18, *p* < 0.001, so that boys showed more life satisfaction than girls. Some country differences were also found in life satisfaction, KW(8) = 576.43, *p* < 0.001. The highest scores in life satisfaction were found in Romania and Spain, while the lowest ones were found in Estonia and Poland.

Table 2 and Table 3 describe the frequencies of playing sports and electronic games by country. The results also showed some significant differences by country in the frequency of playing sports or doing exercise, KW(8) = 277.03, *p* < 0.001, and in the frequency of playing electronic games, KW(8) = 501.03, *p* < 0.001. Concerning the frequency of playing sports or doing exercise, Romania, Malta, and Croatia showed the greatest percentages in playing every day, while Italy and Belgium showed the lowest. Furthermore, Malta, Romania, and Estonia presented the greatest frequencies in playing electronic games every day, while the lowest ones were detected in Spain and Hungary.

### 3.2. Effects on Life Satisfaction by Playing Sports and Electronic Games

The results showed that participants who practiced sports every day (M = 9.01, SD = 1.68) presented the highest levels of life satisfaction, while those who never practiced it showed the lowest levels (M = 8.03, SD = 2.43), KW(5) = 342.41, *p* < 0.001. Furthermore, a significant association was observed with the frequency of playing electronic games, KW(5) = 18.44, *p* = 0.002. Participants who played electronic games every day reported more life satisfaction (M = 8.70, SD = 1.86) than those who never played them (M = 8.46, SD = 2.13). After these separate bivariate analyses, a linear regression analysis was tested to examine the effects of the two variables, i.e., playing sports and playing electronic games, on life satisfaction. Table 4 presents the effects of the frequency of playing sports and playing electronic games on life satisfaction in the total sample and by country and gender. In the total sample, playing sports had a positive effect on life satisfaction, while no significant effect was observed by playing electronic games. Similar effects were observed by gender and country. Playing electronic games only had a small effect on life satisfaction in Hungary, Poland, and Romania. The percentage of explained variance of life satisfaction based on these two leisure time activities was small. Concerning differences by countries, the strongest effect of playing sports on life satisfaction was detected in Italy, while the weakest was found in Poland.

### 3.3. Relative Frequency of Playing Sports vs. Electronic Games and Differences in Life Satisfaction

**Figure 3:**
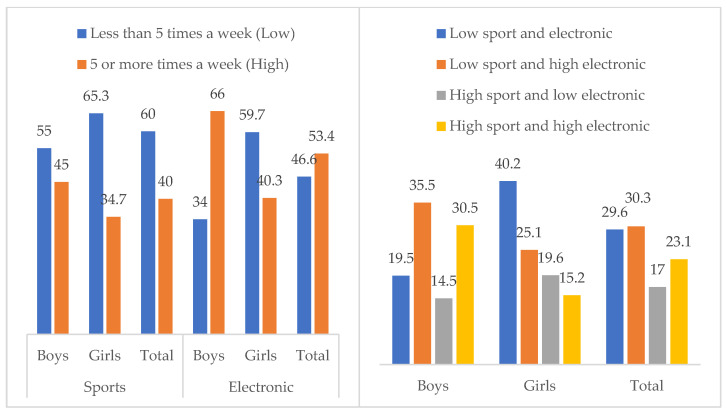
Frequency distribution of the new variables based on high or low practice of sport and use of electronic games, in the total sample and by gender.

The two lifestyles variables were transformed into dichotomic (low level: less than five times a week, high level: five or more times a week), in order to calculate a new variable based on the frequency in both sports and electronic games. Figure 3 describes the frequency distribution in these new variables. A total of 40% of the sample reported a sport practice with a frequency of five or more times a week, with a greater percentage seen among boys, Χ^2^(1, *N* = 10,184) = 110.87, *p* < 0.001, φ = 0.104. Regarding electronic games, more than the half of the sample reported a frequency of five times or more a week, with a bigger percentage in the male subsample, Χ^2^(1, *N* = 10,261) = 679.02, *p* < 0.001, φ = 0.257. Concerning the new variable integrating both habits, the results in the total sample showed that 17% reported a high level of sport practice and a low use of electronic games, while 30.3% indicated a low level of sport practice and a high use of electronic games. Significant differences by gender were also observed, Χ^2^(3, *N* = 10,117) = 751.76, *p* < 0.001, φ = 0.273. The percentage of girls who presented low sport practice and electronic gaming was double the percentage of boys. Furthermore, the percentage of boys with high levels of playing both sports and electronic games was double the percentage in the girls’ sample.

**Figure 4:**
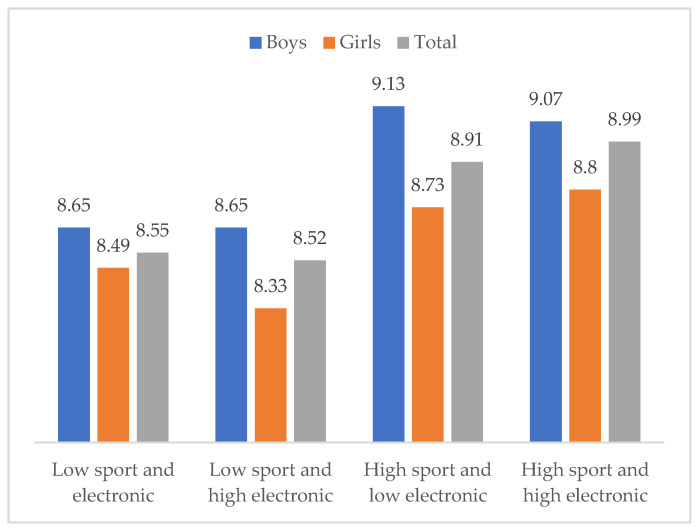
Means in life satisfaction by the relative frequency of sport practice and electronic gaming, in the total sample and by gender.

Finally, differences in life satisfaction were calculated by the levels in the practice of both leisure time activities. Figure 4 shows the means in life satisfaction by the levels in the relative practice of sports and electronic gaming, in the total sample and by gender. In the total sample, KW(3) = 278.01, *p* < 0.001, the highest levels of life satisfaction were observed in the participants with a sport practice regularity of five times or more a week, at any level of electronic games’ use. These differences were consistent in both boys, KW(3) = 157.40, *p* < 0.001, and girls, KW(3) = 86.37, *p* < 0.001. The greatest mean score in life satisfaction was reported by boys who presented a high level of sport practice and low use of electronic games, while the lowest life satisfaction was reported by girls with a low level of sport practice and high level of electronic gaming.

## 4. Discussion

The permanent search for a better quality of life is one of the main challenges facing society in the 21st century, in which children must have the necessary conditions and support to achieve personal and sustainable growth that will allow them to lead a healthy and satisfactory life. Hence, the value of allowing and encouraging children to have access to physical activities that are varied and at the same time relevant to their age and abilities [26], limiting the time they devote to sedentary activities.

Among the main results of the cross-sectional and descriptive study applied to a sample of 10,626 12-year-old boys and girls in selected schools in nine countries from the European Union, there is evidence that contributes to the discussion regarding lifestyles factors that contribute to life satisfaction. In this sense, it is presented as a first aspect of consideration that 40% of the adolescents participating in the study stated that they practiced sports or exercised five or more days a week, surpassing only 13.9% who expressed that they practiced sports or exercised less than once a week, or never. Moreover, the results highlight the presence of significant gender differences in the total sample, with boys reporting more frequent sport practice.

Furthermore, when analyzing the use of electronic games, it is observed that more than half of the total sample of boys and girls (53.4%) reported that they used electronic games five or more days a week, also exceeding the 21% who indicated that their use was limited to less than once a week or never. Significant gender differences were also observed, with boys reporting more frequent use than girls.

In relation to the process of describing the mean and standard deviation of life satisfaction, by country and gender, the study shows a notable satisfaction with life on the part of 12-year-old boys and girls, which does not rule out the presence of data that show differences between the countries themselves.

Sport and physical activity have an impact on the development of children in different physical, psychosocial, and intellectual aspects, promoting more active lifestyles in the present (childhood) and most likely in adulthood [27,28]. Consequently, the research infers that the impact of practicing sports and exercise on children is greater than the use of computers, finding similarities with the approaches in the literature that highlight that leisure and having free time promotes personal satisfaction, influencing both physical and mental well-being [2,5], justifying the priority adopted by many European countries in recognizing the promotion of physical activity as a priority and in the implementation of plans for its greater attraction [24].

In this sense, for the participants of our study, the results suggest that sport practice promotes experiences with a high degree of gratification, free expression of emotions, motivations, values, and benefits [8,9]; furthermore, physical activities and sports performed in leisure time as well as in institutional spaces such as schools can be recognized, in general, as pleasurable, beneficial, and even as generators of well-being [1,24,27]. Hence, the greater impact of the practice of sports and exercise over the use of the computer proves the value of having and enjoying free time and free spaces for self-gratification.

The challenge of consolidating social, sports, and educational institutional policies to encourage physical activity—mainly in the school environment, but also in sports centers via activities that appropriately and responsibly awaken a desire for physical activity—is evident, especially since it relates to a large part of children’s time in their daily lives. At the same time, it becomes necessary to adapt the communication strategies that account for the contributions of physical activity in the daily lives of all people. In addition, awareness of the benefits of physical activity among family members, through training coordinated by the educational establishments themselves, can also provide necessary support.

The recommendations made by the WHO [24,28] suggest that people between 5 and 17 years of age should perform at least 60 min of moderate to intense physical activity daily, making clear that the health benefits of exercising for longer are superior [29]. However, as mentioned, the reality observed by the study shows that of the total number of participants, the average of those who practiced sport or exercised every day or almost every day was below 50% (45% of boys and 34.7% of girls), with differences between countries, where those above the average detected were Romania, Malta, and Croatia. In any case, we would need further information concerning physical activity to expand these conclusions, such as the duration, intensity, or type of physical activity.

The performance of physical activity in any form is directly related to health benefits if it can be performed with regularity, as well as sufficient duration and intensity [5]. Considering the variety of factors that may facilitate or hinder the performance of physical activity, it is important to implement strategies that increase its diversity and quality, offering increasingly suitable spaces and infrastructure to enable access to it [30,31].

The results achieved maintain continuity with those provided by the study applied on a sample of 227,441 young people aged 11, 13 and 15 years, which belongs to 45 countries/regions who participated in the 2017/2018 Health Behaviour in School-aged Children (HBSC) survey [32], regarding the presence of low levels of physical activity. Thus, in light of more recent international studies [33,34,35,36,37,38], research data find persistence and concern regarding the low insufficiency of prevalence towards physical activity by adolescents in different regions of the world, including the European region. In this sense, with a view to discuss with recent results showing a decrease in physical activity and an increase in sedentary activities among most school-aged children during the COVID-19 pandemic [39], we infer the high complexity and permanence that crosses the analyzed thematic.

Considering the characteristics of the information collected in the schools of the selected European countries, the results of this study (in line with previous studies) showed that boys are more active and practice sports/exercise for longer periods than girls [38,40,41,42,43], a situation that has been maintained at the same levels for some time. In this sense, although the aspects that influence this differentiation may be varied, inferring that they may be related to issues related to habits, reasons, and motivations to perform the activity [43], it is a priority to study the aspects that condition this reality [44] and, thus, promote the development of physical activity proposals, especially at school, aimed at the greater participation of girls.

Comparatively, the average use of electronic games every day or almost every day reached more than a half of the study participants, and, comparatively speaking, it was higher than those who practiced sports or exercised every day. This is related to the statement that sedentary activities, such as computer use, are becoming more and more widespread [45,46]. Furthermore, the results confirm the findings [47], which indicate that the greater the use of technology, the lower the levels of physical activity, and that greater exposure to the screen is also associated with effects related to lower physical fitness, low self-esteem, discomfort, and even depression [48,49,50,51]. In fact, a study involving European countries found that prolonged screen exposure may be associated with stress and lower school satisfaction [52]. Conversely, participation in sports activities decreases the likelihood of participating in after-school screen activities [53,54]. Daily computer use, whose effects are very small in relation to life satisfaction, also differs from country to country. Among the countries showing above-average results are Malta, Romania, and Estonia. In addition, like studies with similar results [49], boys (66% indicated a frequency of five days or more a week) reported more frequent computer use than girls (40.3%).

In the same line of a previous study conducted in a country from a different region of Europe [55], a remarkable satisfaction with life on the part of children was observed. Hence, the application of measurements to recognize well-being increases their understanding of their state, being a guide to recognize actions oriented for its improvement [56,57]. In this sense, in line with a recent study [58], a relationship between the use of their leisure time and the satisfaction experienced by children is appreciated. Consistently with the findings provided on the selected countries, where a moderate positive effect on life satisfaction was observed when practicing sports or exercising, there is also evidence that sports and physical activities are associated with an increase in subjective well-being [59], in positive feelings [60], in academic performance [61], and directly and indirectly in the cognitive, emotional, learning, and even neurophysiological domains [62].

The association between low levels of physical activity and an increased use of new technologies, and, consequently, the increased time spent by children in front of a screen, cannot be understood without considering the current society, which is increasingly affected by circumstances that constantly pose new and different threats to well-being in general and to children in particular [63]. Faced with this contextual scenario, it is important to consider the greatest possible involvement of parents and teachers to promote the reduction and adequacy of exposure to the computer screen, in an era where the use of digital technology is so integrated [47,64,65].

In addition, it is concluded that the deepening of factors that have been experienced globally and in a sustained manner during the last few years, such as: the speed of information; the massive degree of access to technological devices; the increase in the use of technologies; the evolution of technology; ways of moving in the context of access to different means of transportation and urbanization [4,66]; added to the consequences of the COVID-19 pandemic, which has resulted in an increase in the use of screens [67,68,69]) pose new and different threats to children’s well-being [63], being some of the reasons why physical activity is relegated in the general population. In this sense, it is important to improve levels of physical activity by promoting, for example, active travel to school by adolescents [70].

Finally, in relation to possible limitations and future lines of research that can be derived from the study, it is necessary to deepen, in a greater variety of regional contexts, the interactions of the practice of physical activity and sedentary behaviors, including computer use, in their relationship with life satisfaction and quality of life. Consequently, considering that low levels of physical activity compromise not only current but also future health [34], and that physical activity is a promising resource for sustainable development [71], it makes sense to conduct research aimed at developing measures that can be used for comparisons between countries, as culturally diverse groups [72], focusing on consulting the children themselves [63]. Moreover, considering the fundamental role that the school plays for the development of children, it is necessary to consolidate it as an institution that stimulates physical activity and sports. Therefore, considering that high levels of screen time can be associated with various psychological and mental health outcomes that are considered unfavorable [73,74], and facing a reality that shows the expansion in recent times of the use of various types of devices, in addition to the computer, it is necessary for future lines of research to consider the incorporation of indicators that deepen the knowledge regarding the new and varied ways of relating to the use of screens [75,76].

Future research should include different ages in the adolescent period, including early, middle, and late adolescents, and controlling for the puberty period, especially in order to obtain a further examination of gender differences in leisure time and well-being. Another limitation comes from the assessment of physical activity, with a unique question with categorical responses. Future research should use more complex measures of physical activity such as GPAQ [77], in order to follow WHO recommendations. The analysis of physical activity needs an examination of the duration, intensity, and type of activity, in order to provide more valid conclusions. Thus, our results have the important limitation of addressing frequency, with just a few response options. Moreover, the response options presented do not allow for an adequate discrimination, with most of the sample indicating the last categories. In the case of the present study, the analyses were performed using secondary data from an important international study, the Children’s Worlds project (www.isciweb.org, accessed 1 August 2022) [78]. With data from this project, a future research line could come from the analysis of differences between countries from different continents, sedentary lifestyles, and their effects on emotional aspects [79], obesity [80], and even on depressive symptoms [81,82,83], which could expand the conclusions about the link between physical exercise and life satisfaction to include diverse contexts and cultures [84].

## 5. Conclusions

Thus, this study presents conclusions that highlight the practice of physical activity as a value of high social and individual impact in the search for people’s own satisfaction with life. Its results are intended to be a contribution to the development of childhood policies, providing reliable information that guides educational and social practices in their ability to consolidate programs that encourage sport and recreation, as well as promote moderate sedentary behavior and, thus, increase the possibility of developing subjective well-being and quality of life in school-age children. The present research showed that in both boys and girls of nine countries from the European Union, physical activity showed a positive effect on life satisfaction, while no remarkable effect was observed by sedentary behavior such as playing electronic games.

## Figures and Tables

**Table 1:** Mean and standard deviation of playing sport or doing exercise, playing electronic games, and life satisfaction, by gender and country.

		Life Satisfaction	
Country	Gender	M	SD
Total	Boys	8.84	1.65
	Girls	8.55	1.93
	Total	8.70	1.80
Belgium	Boys	8.94	1.34
	Girls	8.61	1.69
	Total	8.79	1.53
Croatia	Boys	8.98	1.54
	Girls	8.66	1.89
	Total	8.82	1.73
Estonia	Boys	8.13	2.09
	Girls	8.11	2.17
	Total	8.12	2.13
Hungary	Boys	9.06	1.38
	Girls	8.47	1.92
	Total	8.74	1.72
Italy	Boys	8.78	1.49
	Girls	8.34	1.97
	Total	8.57	1.76
Malta	Boys	8.86	1.79
	Girls	8.71	2.01
	Total	8.80	1.88
Poland	Boys	8.37	2.11
	Girls	7.92	2.45
	Total	8.13	2.30
Romania	Boys	9.44	1.17
	Girls	9.30	1.26
	Total	9.37	1.21
Spain	Boys	9.12	1.47
	Girls	8.90	1.70
	Total	8.87	1.55

**Table 2:** Frequency of playing sports, by country.

	Never	Less Than Once a Week	Once or Twice a Week	Three or Four Days a Week	Five or Six Days a Week	Every Day
Belgium	5.5	6.6	27.1	28.5	14.0	18.3
Croatia	9.2	7.0	14.5	22.5	13.8	32.9
Estonia	4.2	7.4	17.8	29.9	16.4	24.4
Hungary	11.7	12.2	25.1	19.2	12.6	19.3
Italy	8.5	6.2	29.2	30.4	10.3	15.4
Malta	6.5	5.7	21.2	20.0	12.5	34.0
Poland	4.3	6.0	16.8	25.2	18.0	29.7
Romania	7.0	8.8	16.7	14.2	12.3	41.0
Spain	5.2	5.7	22.0	29.1	12.7	25.4

**Table 3:** Frequency of playing electronic games, by country.

	Never	Less Than Once a Week	Once or Twice a Week	Three or Four Days a Week	Five or Six Days a Week	Every Day
Belgium	3.3	7.4	10.2	17.7	16.2	45.1
Croatia	9.0	13.3	12.8	13.4	15.2	36.3
Estonia	6.2	6.9	9.0	11.8	16.4	49.6
Hungary	20.0	14.7	13.1	12.9	11.2	28.1
Italy	11.8	8.4	12.4	14.2	14.5	38.7
Malta	4.8	7.3	8.3	9.0	12.5	58.1
Poland	7.0	11.7	13.7	16.0	14.7	36.8
Romania	12.8	8.6	8.4	8.2	12.7	49.4
Spain	15.0	12.7	16.1	15.3	13.3	27.8

**Table 4:** Linear regression analyses of the effects of playing sports and electronic games on life satisfaction, by country and gender.

				VD: Life Satisfaction	
Country	Gender	F	R^2^	Sports β	Electronic Games β
Total	Boys	55.06 ***	0.022	0.15 ***	0.01
	Girls	44.20 ***	0.018	0.13 ***	−0.01
	Total	115.26 ***	0.023	0.15 ***	0.02
Belgium	Boys	4.38 *	0.014	0.12 *	0.06
	Girls	4.78 **	0.015	0.14 **	0.02
	Total	11.52 ***	0.021	0.14 ***	0.05
Croatia	Boys	8.38 ***	0.027	0.18 ***	−0.02
	Girls	6.89 **	0.021	0.15 ***	−0.04
	Total	16.69 ***	0.028	0.17 ***	−0.01
Estonia	Boys	14.47 ***	0.050	0.23 ***	0.04
	Girls	3.25 *	0.009	0.10 *	−0.04
	Total	14.88 ***	0.027	0.17 ***	−0.02
Hungary	Boys	1.64	0.003	0.08	0.04
	Girls	8.05 ***	0.027	0.17 ***	0.04
	Total	17.44 ***	0.034	0.14 ***	0.12 ***
Italy	Boys	16.57 ***	0.051	0.23 ***	0.06
	Girls	9.56 ***	0.030	0.17 ***	−0.06
	Total	26.10 ***	0.043	0.21 ***	0.01
Malta	Boys	3.13 *	0.011	0.11 *	0.05
	Girls	10.63 ***	0.070	0.25 ***	0.10
	Total	12.10 ***	0.033	0.17 ***	0.06
Poland	Boys	0.67	0.001	0.05	−0.01
	Girls	4.47 *	0.012	0.11 **	0.06
	Total	7.38 **	0.012	0.09 **	0.06 *
Romania	Boys	3.57 *	0.046	0.18 ***	0.11 **
	Girls	5.71 **	0.010	0.11 *	0.03
	Total	17.77 ***	0.030	0.16 ***	0.06 *
Spain	Boys	17.89 ***	0.034	0.19 ***	0.01
	Girls	4.21 *	0.007	0.08 *	−0.05
	Total	18.97 ***	0.019	0.14 ***	−0.01

Note. *** *p* <0.001; ** *p* <0.01; * *p* <0.05.

## Data Availability

Data are accessible on the website www.isciweb.org (accessed on 5 March 2022).
